# Priapism in a Child from Homocystinuria from Methylenetetrahydrofolate Reductase MTHFR (C677T) Mutation

**DOI:** 10.1155/2023/2263341

**Published:** 2023-07-13

**Authors:** Ameer Kakaje, Ammar Fadel, Osama Hosam Aldeen, Othman Hamdan

**Affiliations:** ^1^Faculty of Medicine, Damascus University, Damascus, Syria; ^2^University Hospital Geelong, Barwon Health, Victoria, Australia; ^3^Redcliffe Hospital, Metro North Health, Queensland, Australia; ^4^Department of Haematology, Children's University Hospital, Damascus University, Damascus, Syria

## Abstract

Priapism is a prolonged involuntary erection that can have severe complications if left untreated. Ischaemic priapism is very rare in children and requires urgent intervention to prevent permanent erectile dysfunction and penile shortening. It can be caused by ischaemia in sickle cell anaemia, leukaemia, trauma, drugs, or idiopathy. Homocystinuria is a rare autosomal recessive disorder that can cause hyperhomocysteinemia and hypercoagulability. Very few reports suggested that priapism can be caused by hyperhomocysteinemia, and they were in adults. However, we present the first of such a case to the best of our knowledge of a prepubescent child who only had the MTHFR (C677T) mutation that causes homocystinuria and had ischaemic priapism. A nine-year-old Syrian Arab boy was presented with priapism that lasted for a few hours. Blood tests show normal blood count, film, and haemoglobin electrophoresis. However, prothrombin time, partial thromboplastin time, homocysteine level, and C-reactive protein were elevated. Other coagulation tests were within the normal range. Doppler ultrasonography found decreased cavernous blood flow, and warm 0.9% saline lavage of the cavernosa was indicated and successfully treated the priapism. Genetic testing for the homozygous MTHFR (C677T) mutation was later confirmed, and warfarin was indicated. In conclusion, homocystinuria is very rare in priapism, and this is the first case to report this phenomenon in a child. Ultrasonography in low-income countries is an essential tool that helps identify a wide variety of medical conditions such as priapism and can be successfully managed by aspiration with warm saline.

## 1. Background

Priapism is a painful, prolonged erection of the penis that persists beyond 4 hours despite no sexual stimulation. It is a rare urological emergency that can cause permanent erectile dysfunction and penile shortening [1]. Ischaemic priapism occurs when a thrombus in the corpus cavernosa causes compartment syndrome [1]. Around 65% of causes are found to be sickle cell anaemia, 10% leukaemia, 10% idiopathic, 5% drug-related, and 10% from trauma [2]. Increased levels of homocysteine may predispose to arterial and venous thromboembolic events. This increase may be from homocystinuria which is a rare inherited condition caused by a mutation in the methylenetetrahydrofolate reductase (MTHFR) gene (Rs1801133) and has many variants [3].

In this case, we present a child with priapism from homocystinuria which is to the best of our knowledge the first case to be reported in a child and was treated by corporal aspiration and lavage.

## 2. Case Presentation

A nine-year-old boy presented to the emergency department (ED) with a history of spontaneously painful erection of the penis that started today without a preceding event. It lasted several hours before being presented to the ED. The patient had a fever for 3 days before the presentation. Otherwise, the patient had no other symptoms.

**Figure 1:**
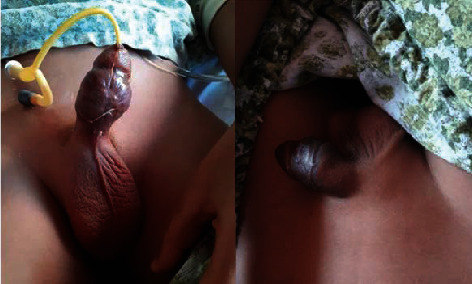
Demonstrating the painful penile erection with and without catheterisation.

On examination, the patient was in pain with an erected penis. The patient was able to urinate but with difficulty (Figure 1); however, he was in good condition and well-grown for his age. He was also succeeding at school. He had no puberty features; Tanner's scale was one. There was no lymphadenopathy, ecchymosis, or jaundice. The eye examination was normal. No signs of abuse were found, and the mother and child denied any history of trauma.

**Figure 2:**
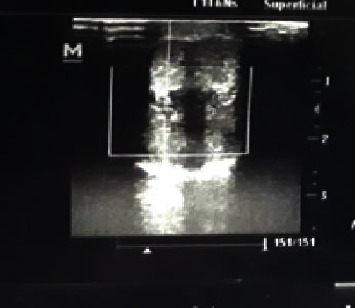
Demonstrating the Doppler ultrasonography with no flow to cavernous arterial flow.

The patient had mild anaemia (9.6 g/dl). The full blood count (FBC) and blood film were otherwise normal. Other blood tests revealed a prolonged prothrombin time (PT) and a moderately elevated CRP. Electrolytes and other blood tests were also normal. Haemoglobin electrophoresis was normal, with no signs of abnormal haemoglobin such as HbS or HbF and normal HbA2 which excluded sickle cell anaemia and thalassemia. Folate, B12, and iron deficiencies were all excluded. Penile Doppler ultrasonography revealed decreased blood flow to the cavernous bodies (Figure 2). Abdominal ultrasound was also normal except for some inflammatory mesenteric lymph nodes, ranging between 1 and 1.3 cm in diameter.

The patient was given IV dextrose and analgesia. An immediate surgical consultation was sought, and an urgent aspiration with warm 0.9% saline lavage of the cavernosa to clear the sludge blood was conducted. There was dark, deoxygenated blood which confirmed the ischemic priapism. This intervention successfully relieved the patient from the priapism.

A full coagulation study was later requested to find the cause of the low-flow priapism while the patient was on analgesics, fluids, and low-molecular heparin which were later switched to warfarin. Protein C (activity) was mildly elevated, while protein S (activity), fibrinogen degradation products, and antithrombin III (antigen) were normal. However, homocysteine was elevated. A molecular study with PCR for hypercoagulability mutations was requested, and the result revealed that factor II, factor V Leiden, and the MTHFR (1298) mutations were normal, while the MTHFR mutation (C677T) result was a homozygous mutant gene. This homozygosity predisposes arterial and venous thrombosis in the presence of additional risk factors [4, 5].

The FBC on the last day of admission was normal with an international normalized ratio (INR) of 2.8, and the patient was discharged on warfarin 0.23 mg\kg daily, and analgesia after the erection and fever were resolved. The patient was well after six months of follow-ups.

## 3. Discussion

Ischaemia is the most common cause of priapism, which accompanies low arterial flow to the corpora cavernosa, resulting in increasing intracorporal pressure and compartment syndrome. It usually spares the glans penis and corpus spongiosum as they have different drainage routes [6].

Homocystinuria is an autosomal recessive disease that leads to marked hyperhomocysteinemia. Some patients may have hyperhomocysteinemia from folate and B12 deficiencies and renal failure [3]. It typically presents with optic lens dislocation, learning difficulties, and marfanoid habitus, and it remains asymptomatic until adulthood [3]. The optic examination was normal, and no features of marfanoid habitus were found. He also had normal folate and B12.

Around half of patients with untreated homocystinuria suffer from a vascular event before 30 years of age, but lowering the homocysteine level significantly reduced the cardiovascular risk [7]. It is speculated that priapism is caused by the increased viscosity of the slow and congested bloodstream which ultimately causes ischaemia [2]. This should be managed by immediate decompression by aspiration until bright blood is seen which indicates that it is oxygenated. Then it should be flushed by using warm normal saline (0.9%) [2]. In our case, this method was used successfully in the patient. The Doppler ultrasound can be used in an acute setting to identify priapism and is readily available in low-income countries.

The metabolism of folate and homocysteine is highly dependent on the MTHFR enzyme. There are 34 mutations identified in MTHFR-deficient patients with homocysteinuria, with *C677T* being a common mutation [8]. The effect of the MTHFR (Rs1801133) genotype and cardiovascular risk is controversial, as studies showed no association between it and stroke, dyslipidaemia, and hypertension [9]. However, there was a higher risk of ischemic heart disease with this genotype [9, 10].

Only a few reports were found for priapism to be caused by homocystinuria in adults [4, 5]. In these two cases, they had persistent high hyperhomocysteinemia for many years, as they were diagnosed after 30 years of age. Other causes of priapism were excluded from our case with a full coagulability study. The high homocysteine with the homozygous mutations of MTHFR is highly suggestive of homocystinuria to be the cause of the priapism. However, this can be coincidental as priapism can not be ischaemic, but these are usually painless and can resolve spontaneously [6].

## 4. Conclusion

Priapism in children is rare and requires urgent management. Ischaemia priapism can be caused by clots such as in homocystinuria which is a rare cause. This is the first case report to the best of our knowledge of priapism in a child, caused by homocystinuria.

## Data Availability

Data will be made available upon reasonable request.

## Abbreviations

CRP: C-reactive protein
ED: Emergency department
FBC: Full blood count
Hb: Haemoglobin
LMW: Low-molecular-weight
MTHFR: Methylenetetrahydrofolate reductase
PCR: Polymerase chain reaction
PT: Prothrombin time
PTT: Partial thromboplastin time.
